# Reasons for Crown Failures in Primary Teeth: Protocol for a Systematic Review and Meta-Analysis

**DOI:** 10.2196/51505

**Published:** 2023-11-01

**Authors:** Stephan Lampl, Deepa Gurunathan, Jogikalmat Krithikadatta, Deepak Mehta, Desigar Moodley

**Affiliations:** 1 Department of Cariology Saveetha Dental College and Hospitals Saveetha Institute of Medical and Technical Sciences, Saveetha University Chennai India; 2 Department of Pediatric Dentistry Saveetha Dental College and Hospitals Saveetha Institute of Medical and Technical Sciences, Saveetha University Chennai India; 3 Smile and Education Centre Wolfurt Austria

**Keywords:** crowns, primary teeth, biological complications, technical complications, survival rates, pediatric, dental, dentistry, teeth, dentists, survival, quality assessment

## Abstract

**Background:**

There is a tendency nowadays to restore large defects in primary dentition with pediatric crowns instead of conventional restorations. Thus, understanding the factors contributing to the survival or failure of dental crowns in pediatric dentistry is essential for optimizing treatment outcomes.

**Objective:**

The primary objective of this protocol is to outline the methodological approach for analyzing data from observational studies and randomized controlled trials to investigate reasons for the failure of dental crowns in primary teeth and to compute their survival and failure rates.

**Methods:**

A comprehensive literature review will be performed in electronic databases, including PubMed (MEDLINE), Cochrane, Embase, and Web of Science. As per predefined inclusion criteria, we will include observational studies (prospective clinical studies) and randomized clinical trials that have an English abstract and involve children aged 1-10 years undergoing crown restorations. Two independent reviewers will independently screen all retrieved records and full-text articles and extract data. The study’s methodological quality will be appraised using suitable tools. Assessments of publication bias will be performed using funnel plots. The findings will be described qualitatively for the systematic review. If possible, a meta-analysis will be performed to estimate failure rates by dividing the number of failures by the total exposure time. A Poisson regression model, assuming constant event rates, will be used to compute 3-year and 5-year survival proportions. The Pearson goodness-of-fit statistics will be used to assess the heterogeneity of the model. A *P* value <.05 will be considered significant. All analyses will be performed using R Statistical software (version 4.1.2; R Core Team).

**Results:**

This systematic review and synthesis aim to assess the survival and failure rates of dental crowns in pediatric dentistry. By following this rigorous methodology, we seek to provide valuable insights into the factors contributing to the success or failure of these restorations. The results of our full review will have implications for pediatric dentists, researchers, and policy makers, helping to improve dental care for children.

**Conclusions:**

This systematic review protocol helps in establishing a thorough approach for reviewing failures in pediatric crowns. By following this methodology, standardization and transparency of the process as well as accountability for the stated methods and outcomes will be ensured. The findings of this review and analysis will provide useful data on the survival of crowns to pediatric dentists and researchers.

**Trial Registration:**

PROSPERO CRD42023442266; https://www.crd.york.ac.uk/prospero/display_record.php?RecordID=442266

**International Registered Report Identifier (IRRID):**

PRR1-10.2196/51505

## Introduction

Apart from maintaining space for permanent teeth, primary teeth serve important functions in mastication and speech development [1-3]. Structural damage to primary teeth caused by caries, bruxism, and other parafunctions, such as jaw clenching and tooth grinding, are relatively common presentations in pediatric dentistry. In 2010, it was shown that untreated caries in deciduous teeth affected 9% of the global population or 621 million people worldwide [4]. Furthermore, the prevalence of bruxism could be in the range of 3.5% to 40.6% among children and adolescents [5]. This high prevalence of caries and structural damages caused by caries and other parafunctions warrant restorative interventions that are safe and effective in the long term [4,5].

Pediatric dental crowns are commonly used as a restorative measure to preserve the tooth's functionality and prevent premature loss. Several materials, such as stainless steel, zirconia, composite resins, and polycarbonate materials, have shown satisfactory properties for fabricating dental crowns [6-8]. Despite advancements in dental materials and techniques, crown failures do occur, leading to potential complications and the need for further interventions [9].

Although secondary caries seem to be a common reason for the failure of these crowns, several other biological and technical factors also contribute to the failure of crowns [9]. Several studies have investigated the outcomes and success rates of dental crowns in pediatric dentistry, but there remains a lack of comprehensive synthesis and analysis regarding the specific reasons for crown failure. Therefore, conducting a systematic review and meta-analysis to pool and analyze available evidence on the reasons for crown failure in pediatric patients will provide valuable insights into this important clinical concern.

The objective of the systematic review and meta-analysis proposed in this protocol is to comprehensively analyze the reasons for the failure of crowns in primary teeth. By synthesizing existing evidence, we aim to identify the common causes of failure, assess their prevalence, and explore potential risk factors associated with crowns in primary teeth.

## Methods

### Protocol Registration and Reporting Information

This protocol is registered in the PROSPERO database (CRD42023442266) and has been written according to the MOOSE (Meta-Analysis Of Observational Studies in Epidemiology) and the PRISMA-P (Preferred Reporting Items for Systematic Reviews and Meta-Analysis Protocols) guidelines [10,11].

### Data Sources, Search Terms, and Search Strategy

**Figure 1:**
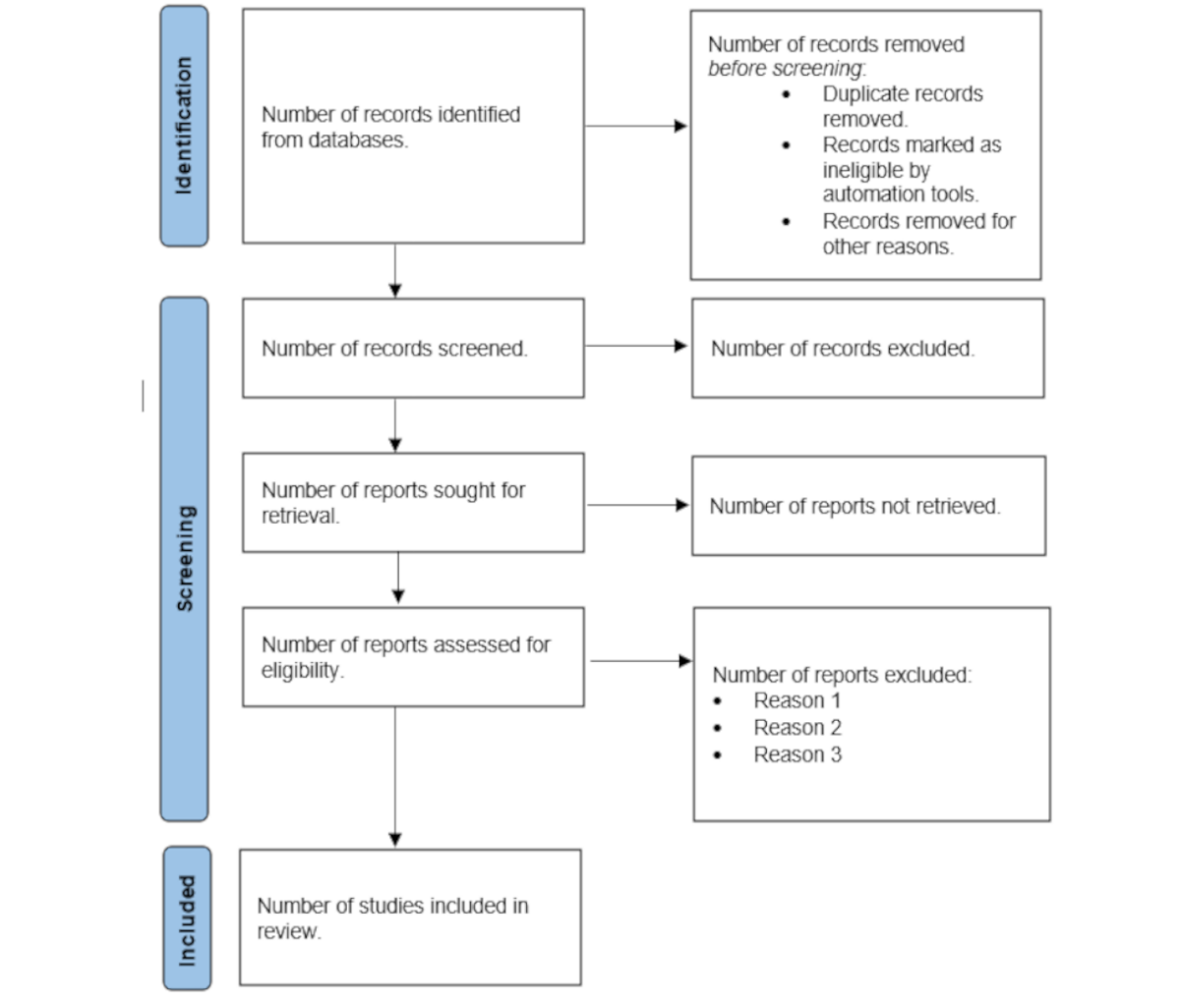
Conceptual PRISMA (Preferred Reporting Items for Systematic Reviews and Meta-Analyses) diagram for identification of studies via databases and registers.

Under the supervision of the principal investigator, we will conduct a systematic search of electronic databases, including Cochrane, Embase, PubMed (MEDLINE), and Web of Science to identify relevant articles published up to the date of our search. The search strategy will be developed using appropriate keywords and Medical Subject Headings terms related to “child,” “children,” “toddler,” “crowns,” “pediatric dentistry,” and “treatment failures.” Additionally, we will manually search the reference lists of selected articles and relevant systematic reviews to identify any additional studies. The searches will not be limited by historical time constraints. The search strategy will be considered adequate to reduce the risk of selection and detection bias. The search results will be exported to Zotero, where duplicates will be excluded. Included studies will be manually searched to select other relevant studies. A PRISMA (Preferred Reporting Items for Systematic Reviews and Meta-Analyses) flow diagram (Figure 1) will be reported to describe the study selection process.

### Inclusion and Exclusion Criteria

We will include clinical studies, randomized controlled trials (RCTs), and prospective longitudinal studies (nonrandomized clinical studies and cohort studies) that meet the following criteria: (1) evaluate crown restorations in pediatric patients aged 1-10 years, (2) report reasons for crown failure or complications, and (3) have abstracts available in English. Qualitative interviews, quasi-experimental studies, single-case studies, and series of single-case studies will be excluded. Furthermore, studies without sufficient data on the survival of crowns or duplicate publications will be excluded. Secondary caries, endodontic complications, and periodontal pathology will be considered biological complications in this analysis. The technical complications to be considered in this analysis will include fractures of the crown (not the tooth itself), loss of retention, debonding, and occlusal wear. Data based on conference abstracts and dissertations will not be included.

### Data Extraction and Analysis

Two independent reviewers will screen the articles for eligibility; they will extract data and assess the quality and risk of bias of the included studies. Any discrepancies will be resolved through discussion or consultation with a third reviewer. The extracted data will include study characteristics, sample size, patient demographics, types of materials, reasons for failure, and reported complications. A narrative synthesis will be performed to summarize the findings, and if feasible, a meta-analysis will be conducted to calculate pooled estimates of the prevalence of restoration failures in pediatric dentistry.

### Assessment of Methodological Quality

The risk of bias in the included RCTs will be systematically assessed using the RoB 2 (Risk of Bias 2) tool developed by the Cochrane Collaboration [12]. Using this tool, 2 independent reviewers will evaluate the various domains of bias in RCTs, including randomization, allocation concealment, blinding of participants and personnel, blinding of outcome assessment, incomplete outcome data, selective reporting, and other sources of bias. Each included RCT will be assessed for risk of bias in accordance with the domains specified by the RoB 2 tool, and an overall risk of bias judgment (low, some concerns, or high) will be assigned to each trial [12]. Furthermore, a checklist proposed by Moga et al [13] will be adapted for assessing the risk of bias in prospective clinical studies. Only studies with a moderate or low risk of bias will be included in the current analysis. Although this tool was originally developed for case series, a modification of this tool has been adapted previously for assessing the risk of bias in studies that have a missing comparator [14]. Using this tool, seven domains will be assessed for risk of bias (Table 1) and an overall risk of bias judgment (high risk of bias, serious risk of bias, low risk of bias, or no information) will be assigned to each study [13,14].

**Table 1:** Risk of bias domains to be assessed for prospective clinical studies. Assessments will be reported as high risk of bias, serious risk of bias, low risk of bias, or no information [13,14].

Domains	Assessment item
Domain 1	Study design
Domain 2	Study population
Domain 3	Interventions
Domain 4	Outcome measures
Domain 5	Statistical analysis
Domain 6	Results and conclusion
Domain 7	Competing interests

### Assessment of Publication Bias

To enhance the transparency and accuracy of the findings, assessments of publication bias (less likelihood of publishing studies that failed to show statistical significance) using funnel plots will be undertaken in the evaluation of the survival and failure rates of dental crowns in pediatric dentistry. If present, publication bias will be reflected as an asymmetry in the funnel plot [15,16].

### Meta-Analytic Approach

For the purposes of quantitative analysis, survival will be defined as a number of crowns that were in situ, regardless of complications (technical and biological), which include secondary caries, marginal integrity, marginal discoloration, and loss of anatomical form along with surface roughness, endodontic complications, loss of retention, and fractures. Failure rates resulting from biological and technical failures will be calculated by dividing the number of failures by the total exposure time. Exposure time for each included study will be calculated by taking the sum of exposure time for all fixed dental prostheses. A Poisson regression model will be used to analyze the calculated rates. Survival proportions for 3 years and 5 years will be estimated with an assumption of constant event rates. The Pearson goodness-of-fit statistics will be used to assess the heterogeneity of the model. A *P* value <.05 will be considered significant. All analyses will be performed using R Statistical software (version 4.1.2; R Core Team).

## Results

### Overview

The search and screening for the systematic literature review are anticipated to be finished in October 2023. Data extraction, quality appraisal, and subsequent data synthesis began in October 2023. The review is expected to be completed by November 2023, and attempts to publish the study results will be made in December 2023.

### Protocol Amendments

If this protocol is substantially amended after an initiation that may impact the conduct of the study (including eligibility criteria, study objectives, study design, study procedures, and analysis), then an amendment will be agreed upon by all collaborators prior to the implementation and will be documented in a later report.

## Discussion

### Key Findings

This planned systematic review and meta-analysis will systematically identify the common causes of failure, assess their prevalence, and explore potential technical and biological factors associated with crown failures in pediatric patients. Furthermore, a meta-analysis will be attempted, if feasible; a Poisson regression model, assuming constant event rates, will be used to compute 3-year and 5-year survival proportions. The findings of the systematic review and meta-analysis will assess the quality of available studies and provide important insights for methodological aspects of future research. Thus, the findings of this review and its quantitative assessments could inform researchers and clinicians.

### Comparison to Previous Research

Chisini et al [9] reported a systematic review on survival and reasons for failure of primary teeth restorations [9]. This systematic review included data from 31 studies conducted between 1996 and 2016 [9]. However, this review looked at all restorative materials used in pediatric dentistry and was not restricted to crowns on primary teeth. Apart from this study, a systematic analysis of the technical and biological factors affecting the survival of crowns for primary teeth has been scanty. Our review will analyze pediatric crowns only and, therefore, will add valuable insights for understanding the technical and biological factors contributing to the success or failure of crowns for primary teeth.

### Limitations

Although it has been suggested that researchers must include unpublished literature in meta-analyses and systematic reviews, the inclusion of data from unpublished studies can itself introduce bias [17]. Thus, data from unpublished studies and non–peer-reviewed literature (eg, reports) will be excluded. However, assessments of publication bias will be performed using funnel plots [15,16]. Despite this potential limitation, the findings of the proposed systematic review and meta-analysis will contribute to the existing body of knowledge by identifying common causes of failure, highlighting potential risk factors, and guiding future research and clinical practice to improve the success rate of crowns in children with structural damage of the primary teeth.

### Conclusions

This systematic review and meta-analysis protocol is designed to provide a comprehensive assessment of the common causes of crown failures in primary teeth, explore associated technical and biological factors, and estimate survival proportions over 3-year and 5-year periods. Although building upon the existing body of knowledge, the proposed study distinguishes itself by focusing on the unique challenges and intricacies of crown failures in primary teeth. By systematically identifying factors influencing crown outcomes, our findings will inform dentists in clinical practice about possible causes of pediatric crown failures, helping to minimize these potential failures. This study will also guide future research efforts to improve material properties to increase the clinical longevity of these pediatric crowns.

## Abbreviations

MOOSE: Meta-Analysis Of Observational Studies in Epidemiology
PRISMA-P: Preferred Reporting Items for Systematic Reviews and Meta-Analysis Protocols
PRISMA: Preferred Reporting Items for Systematic Reviews and Meta-Analyses
RCT: randomized controlled trial
RoB 2: risk of bias 2

## Data Availability

All data generated or analyzed during the systematic review and meta-analysis proposed herein will be made available as supplementary files as part of any forthcoming publications.
